# Mining and analysing online social networks: Studying the dynamics of digital peer support

**DOI:** 10.1016/j.mex.2023.102005

**Published:** 2023-01-07

**Authors:** Jasmina Rueger, Wilfred Dolfsma, Rick Aalbers

**Affiliations:** a Business Management & Organisation Group, Wageningen University, Wageningen, the Netherlands; b Department of Business Administration, School of Management, Radboud University Nijmegen, Nijmegen, the Netherlands

**Keywords:** Online data mining, Social science research methods, Online community data, Mixed-methods research, Internet research, Social media, Online interaction data, Social network analysis, Sentiment analysis, Virtual communities, Online social network mining and analysis

## Abstract

In recent years, the rapid growth of user-generated content has led to much research evaluating the patterns of online information exchange. These studies demonstrate that online communities are valuable data sources which provide rich, longitudinal data that would otherwise be difficult, if not impossible to access. Given the increased research interest, mining and analysing online social networks has become an important research domain, encompassing a variety of approaches. To analyse the large number of observations commonly found in online communities, we propose to first mine the data using a so-called Webscraper and then combine Social Network Analysis (SNA) with Sentiment Analysis to explore both content and relationships. The hands-on approach described in this article is targeted at researchers without a background in technical disciplines. Instead of focusing on some of the specific algorithms that facilitate the mining and analysis of online data, we describe how to use and combine out-of-the-box solutions to collect and analyse the online network data. Moreover, we document the steps taken and present important lessons learnt throughout the process of collecting and analysing data from an online health community with 108,569 registered users who contributed to 197,980 discussions with a total of 484,250 replies.

In sum, our method proposes to:

• Extract all relevant data from an openly accessible online community using a Webscraper.
• Determine and visualise the relationships between users and the properties of the social network as a whole using Social Network Analysis.
• Conduct Sentiment Analysis to detect the emotional tone of the online contributions, and to possibly infer further variables from the text such as the personality characteristics of users.

## Graphical abstract

**Figure:**
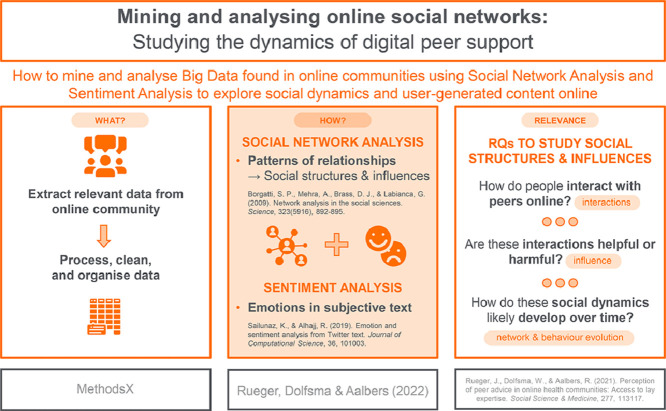

Specifications table

**Table:** 

Subject area:	Psychology
More specific subject area:	Organisational behaviour, social psychology
Name of your method:	Online social network mining and analysis
Name and reference of original method:	Below, we listed some studies that use a similar approach. However, besides some considerable differences in approach, these studies are for researchers with an extensive technical understanding and do not focus on advice/support networks (as commonly found online):Chakraborty, K., Bhattacharyya, S., & Bag, R. (2020). A survey of sentiment analysis from social media data. IEEE Transactions on *Computational Social Systems*, 7(2), 450–464.Habibi, M. N. (2019). Analysis of Indonesia Politics Polarization before 2019 President Election Using Sentiment Analysis and Social Network Analysis. *International Journal of Modern Education & Computer Science*, *11*(11).Fornacciari, P., Mordonini, M., & Tomaiuolo, M. (2015). Social network and sentiment analysis on Twitter: Towards a combined approach. *KDWeb* (pp. 53–64).
Resource availability:	R and various R packages, Gephi; possibly an additional dictionary such as LWIC to analyse the text

## Method details

In recent years, the rapid growth of user-generated content has led to much research evaluating the circumstances and patterns of online information exchange (e.g. [9,^21^,23]). These studies demonstrate that online communities are valuable data sources that can help social scientists better understand the dissemination of information, such as knowledge, experiences, and opinions [16]. For instance, by studying online networks and determining when and how individuals seek or provide information, new and challenging questions may arise about individual and group behaviour.

The Internet has made social contact with a large number of people faster and more convenient, regardless of geographical distance. Consequently, online communities provide rich longitudinal data, reducing issues commonly encountered in other research methods, such as low response rates or self-reporting bias. Moreover, by analysing the interactions of users on online communities, researchers are able to gain insights into data that would otherwise be difficult or even impossible to access. First, it allows researchers to study a larger and more diverse population than would be possible using other methods. Second, online data are often more accurate and detailed than data collected through other means. Finally, online data can be used to study behaviour over time, which can provide valuable insight into how social relationships form and evolve.

Although some online communities are general social networks, many cater to a specific area of interest, such as software development, sports or health and wellness (e.g. [[2], [22], [24], [26], [38]]). As such, online communities provide a platform for digital peer support by allowing people to engage with like-minded people to share knowledge, ideas, and possibly emotional support [[22], [35]]. In the past, different studies have shown that digital peer support can take many forms, such as asking and answering questions, providing feedback, or simply sharing knowledge and experiences [13,25]. In addition to informational support, members of online communities can also exchange emotional comfort and understanding with others who are struggling with similar issues [35]. To give an example of how to study digital peer support using the method proposed in this article, we would like to refer to our own work [28] as well as that of others who have presumably used similar methods to gather and analyse their online community data.

Given the increased interest in researching online communities, mining and analysing online social networks has become an important research domain, encompassing a variety of approaches. For example, Social Network Analysis (SNA) is a popular lens for researchers seeking to analyse the large number of observations commonly found in online communities. However, effectively using data collection and analysis approaches such as Data Mining and SNA for research purposes usually requires advanced technical understanding. Despite the growing body of research and documentation, this may be a barrier for less technically inclined social scientists who would like to use online data for their studies on, for instance, individual and group behaviour.

Unlike other more technical descriptions published previously, this article is intended to address this gap by being targeted at researchers without a background in Computer Science or other technical disciplines. Instead of focussing on algorithms for data mining and analysis, we will describe how to collect and analyse data found in online communities using readily available software solutions. We will elaborate on the steps taken and present important considerations and insights discovered throughout the process. Furthermore, we will discuss the different methodological challenges that social scientists may face when exploring online social network data. The considerations we will address are not limited to technical issues; instead, they include important strategic choices such as how to find a suitable data source or operationalise variables that optimally align the data set with the research question(s). Throughout this article, we will present potential research questions that researchers using the described method could pursue. Lastly, we will critically discuss some of the ethical implications when using online data, as well as the limitations of our method.

### Exploring social networks in online communities

A social network is a set of actors together with a set of ties that represent some kind of connection between pairs of actors. In online social networks, actors are typically people (or organisations), and ties are established through digital interaction, such as messages, follows, and likes. The ties can be positive (friendship, support and admiration), negative (hate, aggression, and violence), or neutral. Collecting social network data means capturing the presence or absence, and possibly the strength, of relationships between pairs of actors [7].

Previous social network studies have provided some insight into how online communities can be used to maintain existing social relationships and build new, entirely virtual relationships. Several studies comparing offline and online relationships have found that social bonds maintained online can be as trusting and supportive as those involving face-to-face interactions [18].

Online communities with threaded discussions such as question-and-answers (Q&A) forums are an easily accessible source of Internet-based communication data. To date, several studies have used ”who replies to whom” data, which evaluates the messages exchanged between individuals from their recorded interactions, to create a communication network (see [17,^19^,27]). User interactions, such as the response of one user to another, can be used to define dyads. When two nodes interact with each other frequently, they are considered to have a strong tie. Alternatively, when two nodes interact with each other infrequently, they are considered to have a weak tie.

In addition, many online communities allow users to create a profile to display information about themselves. Although data from online communities are usually anonymous, researchers can use details such as age, gender, or interests to further characterise actors and their network ties. For example, profile information can help to determine the level of homophily between two nodes or a group of actors.

Lastly, the text of the messages posted in an online community provides insight into the relationships individuals form and maintain, as well as important topics that are being discussed. Most simply, text can be counted for indications of engagement by considering the frequency or length of messages. Alternatively, posts could be read, coded, or text mined for keywords, common phrases, and tone to better understand the kinds of relationships and topics prevalent in the community.

Recent evidence suggests that it is possible to accurately infer personality characteristics from text information based on the type of words an individual expresses [5]. Using Natural Language Processing techniques, software tools such as *Linguist*ic Inquiry and Word Cou*nt*, more commonly known as *LIWC*, are widely used to measure emotion, cognitive styles, and social processes based on word use [10,^32^,39]. A seminal study in this area is the work of Holtgraves [20], who found significant correlations between personality traits such as extraversion, neuroticism, and agreeableness and certain LIWC categories such as frequency of personal pronouns, negative, and positive emotion words.

### Longitudinal social network and text analysis

When conducting research with online communities, there are two main types of data analysis that can be performed: social network analysis and text analysis. While social network analysis is concerned with the social ties people establish, analysing the text the people share with each other in these relationships allows us to better understand not just the presence but also the purpose and implications of such relationships. Together, both approaches can thus be used to examine the relationships and behaviour of community members and how they may change over time.

Online community data provide a wealth of social and behavioural information that can be analysed in a number of ways. Two of the main types of data analysis that can be conducted and therefore addressed in this article are social network analysis and text analysis. Social network analysis looks at the relationships between people within the community, while text analysis, such as sentiment analysis, focusses on the topics that people discuss and how they feel about them. Both relationships and topics are likely to change over time, so longitudinal data are particularly valuable for this type of research.

Looking at how relationships and behaviour co-evolve creates new opportunities for future research. For example, does increased and boundary-spanning social interaction lead to more diverse topics being discussed? Do certain topics become more popular as the community grows? By understanding how the various elements of an online community interact with each other, researchers can gain a better understanding of how these communities evolve over time.

## Data collection

**Fig. 1:**
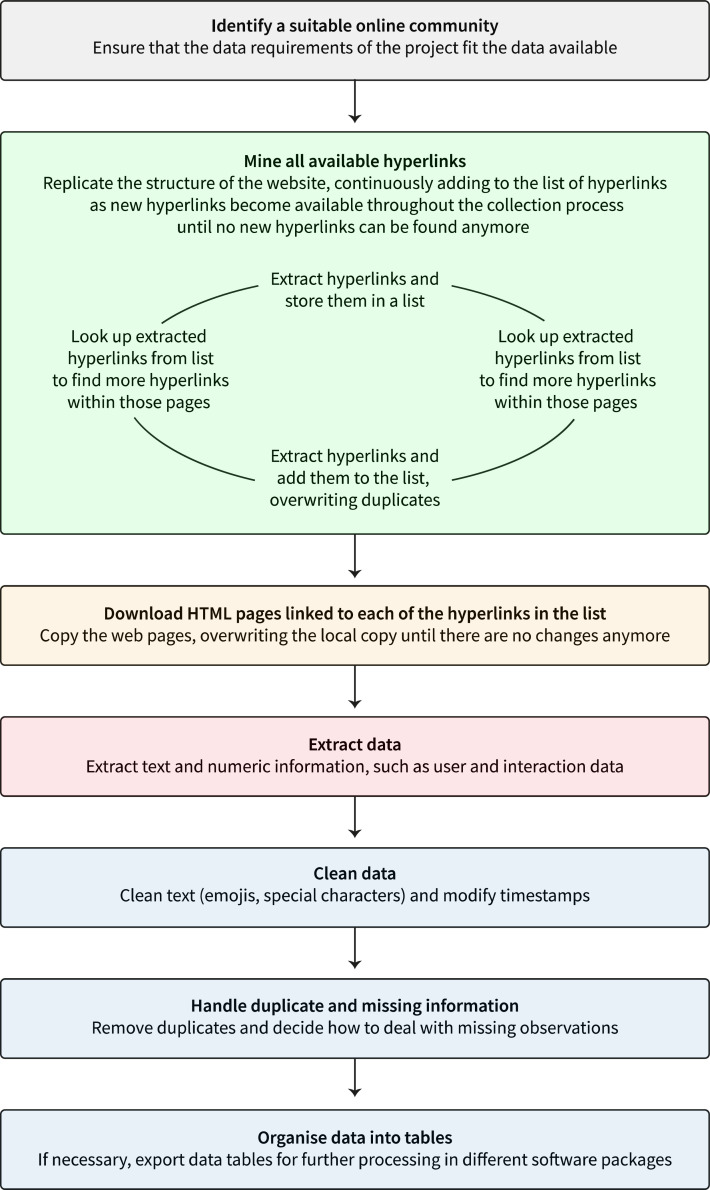
Workflow to extract data from the online community.

The first part of the method described in this article is concerned with the data collection phase which takes place before the second phase, data analysis. To collect data from online communities, researchers must identify the community or communities they want to study. In this section, we will discuss how to select an online community to study and how to collect data from that community. The steps taken during the data collection phase as illustrated in Fig. 1 are: (1) select a suitable online community, (2) extract all relevant hyperlinks, (3) download and mirror web pages locally, (4) extract data from web pages, (5) process and clean text and numeric information, (6) handle duplicate and missing information, and (7) organise the data into tables that are suitable for subsequent analysis in R or a different software package, such as SPSS, STATA or MS Excel.

### Required software packages

There are a number of different software programmes that can be used to collect and analyse data from online social networks. Although we recommend using R to collect and analyse the data, the same principles can be applied to other programming languages and software packages, such as Python or SAS. Our method was tested with R Version 3.6 and RStudio Version 1.0.2.

To collect and analyse the data, we recommend using a variety of R packages, such as *rvest* for scraping the data, *lubridate* for timestamps, and *dplyr* to manipulate and clean the data. To perform social network analysis, we recommend using a combination of the R package *igraph* and*Gephi. igraph* is a powerful R package that can be used to analyse large social network data with complex measures. *Gephi* is a free and open source software package that offers good integration with R and is commonly used to visualise large social network data.

### Selecting an online community

When selecting an online community to study, it is important to consider the purpose of the research project and the type of data that will be most useful in answering the research question. Although the specific requirements for conducting a study of online communities vary depending on the focus and context of the research, there are some general criteria that all studies should meet. First, it is important to define the type of community to be analysed. Is the focus on a specific website or forum, or is it a more general analysis of social media usage? For example, if the aim is to understand how people form and maintain relationships in a specific context, then a community that is focused on social interaction, such as a forum, would be a good choice. If the focus is on understanding the topics that people discuss and their opinions about those topics, then a community that is focused on sharing information, such as a news site or online review site, might be a better choice.

Second, once the type of community has been selected, the next step is to choose a specific community to study. There are several factors to consider when making this decision, such as the size of the community, the level of activity, and the types of relationship that are formed within it. For example, if the goal is to study how relationships change over time, then a large community with a high level of activity would be a good choice, as there would be more data to work with and more opportunities to see how relationships evolve. Furthermore, online communities may be moderated by professionals or volunteers to improve the quality of online discourse. While peer-led communities may self-regulate and users are free to express their opinions, expert-led communities seek to ensure that only correct and helpful information is shared by removing any content that does not match the predetermined protocols, such as potentially harmful, offensive, or threatening content [4,15]. Besides removing content, moderators may also be able to suspend accounts temporarily or permanently. When choosing a particular community to extract and analyse data from, it is thus important to consider the degree of moderation present in the community. For example, a discussion forum that has little to no moderation may contain more misinformation and have different group dynamics than one with stricter standards enforced by active moderators. However, the presence of moderators may not only influence user behaviour in a positive manner. For instance, Griffiths et al. [15] found that users could feel limited and become annoyed by 'faceless moderators' who may remove information users do not believe to be incorrect or otherwise violating the terms of use.

Third, researchers should consider the demographic profile of the users they wish to study. Are users primarily young adults or is the community made up of a mix of ages? What is the gender balance of the community? And where are most of the users located? Knowing the answers to questions such as these can help researchers identify groups within the online community. This step is particularly important if the study is intended to make comparisons between virtual and offline communities.

Finally, researchers should identify any measures they plan to use to collect data on user interactions and behaviour within the community. By carefully defining these criteria before collecting data, researchers will be better equipped to obtain accurate and reliable data that can be used to answer their research question. For example, if the goal is to study the formation of online relationships, then measures such as frequency of communication or number of friends might be important to collect.

**Table:** 

**IMPORTANT CONSIDERATIONS** • Is the focus on a specific context or is it a general analysis of social media usage? • How big should the community be? And how active should the users be? • What types of relationship do users establish with one another? • What demographics (e.g., age, gender, location, education level) are intended for the study's participants? • What measures of interactions and other behaviour will be analysed? Can these measures be inferred?

### Extracting all relevant hyperlinks

The most common method to extract online data is to use an existing tool, such as a *Web crawler*, to automatically collect data from the target community. This is the most efficient way to collect data, but it has some limitations. First, not all online communities can be easily crawled, as some require a login, which complicates the crawling process. Second, even if an online community can be crawled, the data that are collected will be limited to what is publicly available on the website. This means that private messages and other types of data hidden by platform operators or users will not be accessible.

To start extracting the online data, the first step is to create a *seed list* of threads (online discussions) or members. This could be all threads in a certain group or the most active and influential members of the community. The so-called *seed list* is a directory of web pages that is used to start the web crawling process. As the Web crawler visits each web page in the seed list, it identifies all the hyperlinks on each page and adds them to the list of URLs to visit. This process is repeated recursively until all relevant hyperlinks are collected.

### Downloading and mirroring web pages locally

Once the seed list has been generated, the next step is to download and mirror the web pages locally. This can be done using a tool such as *GNU wget*, which is a free command-line package used to retrieve HTTP and HTTPS website files and mirror them locally. This step is important because downloading the pages allows the researcher to have a local copy of the data, which can be used for offline analysis. Furthermore, by downloading the pages, the researcher can be sure that they have a complete copy of the data and that no data is lost due to changes made to the online community, such as threads or profiles being deleted.

Furthermore, *wget* can overwrite existing files, skip files that have not changed, and only download the latest changes. This is especially useful for websites that change frequently, such as active online forums. With each iteration of replacing files that have changed, for example, when a user adds a new question or answer, the delay between the downloaded and online files becomes increasingly smaller. Eventually, by downloading the latest changes until there is no delay, researchers can ensure that they have a snapshot of the community at a particular point in time without missing any changes that occurred during the hours or days it took to collect the data.

**Table:** 

**RECOMMENDATION**When downloading large amounts of data, it is often advisable to run multiple instances of the Web crawler at the same time. This maximises computational power and can help reduce the overall crawling time. However, it is important not to have too many instances running at once, as this can lead to performance issues or slow down the servers of the provider of the online community. To prevent their servers from being overloaded with automated requests, online communities often have rate limits in place. Therefore, it is important to check the terms of service of the online community before starting to crawl to avoid being banned.

### Extracting data from the downloaded web pages

Once the Web pages have been downloaded, the next step is to extract the data of interest from the HTML pages. This can be done using a tool such as rvest, which is an R library that extracts data from HTML pages using CSS selectors. CSS selectors are used to define what elements of a website should be styled in a certain manner. For instance, they might be used to define the colour of a certain object, such as a heading that is visible on a website. We can use the CSS selectors to ’select’ the elements that display the content we want to collect. By automatically extracting data from website elements, such as the title of the thread, the number of replies, and the text of the posts, using CSS selectors and rvest, researchers can save them in a format that is easier to work with, such as tables. In addition, by extracting only the data of interest from the web pages, time and computational resources can be saved that would otherwise be spent on parsing and working with irrelevant data.

When extracting data from HTML pages, it is important to consider the structure of the data. For example, in a forum thread, there may be multiple posts, each with its own title, text, and number of comments. To save these data in tabular format, it is important to consider how the data should be organised. For example, in the case of an online discussion forum, it would be helpful to create a row for each post with the title, text, and number of replies in separate columns. This would allow researchers to easily filter and analyse the data to, for example, only evaluate the most recent posts or those with the most replies.

It is also important to consider how the data will be analysed. For example, if the data are used for text analysis, it might be helpful to extract only the text from the posts and not all the other HTML tags. Similarly, to be able to use the data for social network analysis, it would be advisable to extract the user ID of each post to later construct a network of users.

**Table:** 

**RECOMMENDATION**Once the data have been extracted, it is important to rule out discrepancies between the extracted data in the CSV file and the original data in the HTML pages. This should be done by checking a random sample of a variety of entries in the CSV file and comparing them to the original data.

### Processing and cleaning data

In order to analyse the data collected from the HTML pages, it will likely need to be processed and cleaned. For example, posts in online discussion forums can contain hyperlinks to other websites, emojis, and other irrelevant information. To make data easier to work with and to remove irrelevant or invalid data, it is important to process and clean them.

For text data, it is recommended to use a tool such as R with its extensive range of packages for text and natural language processing. R packages such as stringr can be used to clean the data by removing special characters and stop words, which are common words that do not contribute to the meaning of the text, such as ’the’, ’a’, and ’is’. Additionally, text data can be stemmed or lemmatised, which means that words are reduced to their base form or stem, such as ’running’ being reduced to ’run’. This can be helpful for text analysis, as it reduces the number of different word forms that need to be considered when searching for specific words. If the data consist of timestamps, it might be helpful to convert the timestamps into a format that is easier to work with, such as the number of days since the start of the data collection. This can be done using an R package such as *lubridate* [37]. Furthermore, when plotting graphs, it can be helpful to have the dates clustered as weeks, months, or years.

**Table:** 

**RECOMMENDATION**Emojis and hashtags are often used on social networks to express emotions or group similar posts. However, they can also add noise to the data if they are not used as part of the analysis. For this reason, it is important to consistently interpret emojis or hashtags found in the data or remove them prior to analysis.

### Handling duplicate or missing information

It is also important to check the data for missing and duplicate values. Duplicate values can be easily filtered using R's *dplyr* package. For example, depending on the structure of the data source, it is possible that some posts may appear in different subject areas or discussions. If there are missing values, it is important to decide whether they should be imputed or removed from the data. Imputation is the process of replacing missing values with substituted values, such as the mean or median of the remaining values. However, this should only be done if the number of missing values is very small, as imputation can lead to bias in the data.

### Organising the data set

Once the data have been processed and cleaned, it is important to save them in a format that can be easily read by the chosen software. Researchers may choose to use different software for different types of analysis, such as R, SPSS or STATA for statistical analysis, Gephi or UCINET for social network analysis, and Microsoft Excel or Tableau for data visualisations.

For this reason, it is important to save the data in a universal format that can be read by all applications. The CSV (Comma Separated Values) format is a good choice for tabular data, as it can be read by most software, including text editors and commonly used statistical applications. Furthermore, it is possible to use a separator other than a comma. This is helpful if the data contain commas, as these would be interpreted as separators (end of column) rather than as part of the data. Similarly to the end of columns, the end of rows can be signaled with different delimiters such as \n, \r or \r\n. Especially when mining user-created information, \t (tab) and \r\n are suitable separators and delimiters, as they do not naturally occur in the text.

**Fig. 2:**
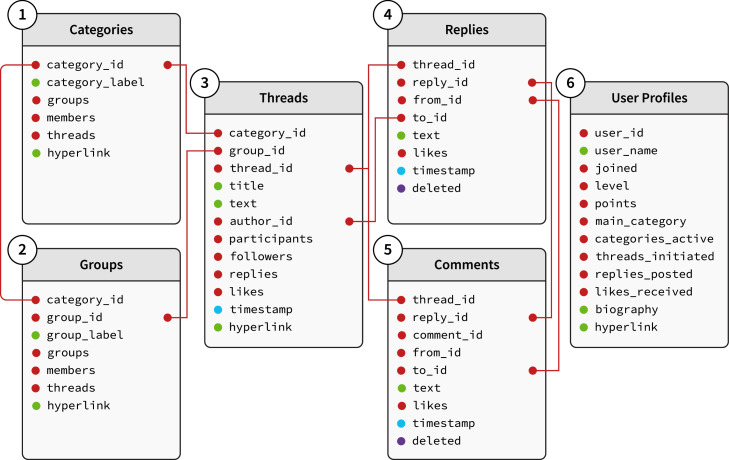
Data schema.

Once saved in CSV format, the data should be organised in a way that will make the analysis more convenient and consistent. For example, unique identifiers for each discussion thread, post, and user should be created if they are not already present in the original data. These IDs can then be used to link different data tables. An example of how the different data tables might be connected to each other is illustrated in Fig. 2. The exact tables will depend on the data source, but, at least for an online discussion forum, will typically include: (1) categories, (2) subcategories, (3) threads, (4) replies, (5) comments, and (6) user profiles. Each of the tables has at least one ID or column that can also be found in another table. For example, the ’replies’ table has a column with the ID of the user who wrote the original post (to_id), which contains the same user IDs as the column author_id in the ’threads’ table.

**Table:** 

**RECOMMENDATION**In addition to the variables extracted from the online community, it might be helpful to create new variables, such as means or sums of posts or users per thread. When adding new variables, researchers should diligently document the process to replicate the results.

#### Categories

Many online communities, particularly online discussion forums, are organised around specific categories. Categories are usually predetermined by the online community administrators, but in some cases, they are created by the users themselves. This grouping of interests makes it more convenient for users to find relevant discussions and connect with others who are dealing with similar issues. For example, when we used this method to collect data from an online health community, we collected data from 32 different categories of health symptoms and illnesses, such as *Cancer, Pregnancy* and *Skin & Nails*.

#### Threads

A thread is a sequence of posts on an online discussion forum that are all replies to the same original post. In most online discussion forums, when creating a new question (i.e., initiating a new thread), the initiating user is required to select a category for their question. In some communities, users may be able to categorise a thread as being part of two or more different categories. For example, a thread with a question about skin cancer might be categorised as both *Cancer* and *Skin & Nails*.

#### Replies and comments

When users reply to a thread, they are essentially creating a new post that is linked to the original thread. In many online communities, users can also leave comments on a thread. These comments are usually shorter than replies and are not intended to contribute to the discussion, but rather to give an opinion on a specific reply. Often, the user who posted the original question uses the ’comment’ function to respond to the replies of other users. Most online communities also have ’like’ buttons that allow users to show their appreciation for a particular reply or comment.

#### User profiles

In most online communities, users are required to create a profile that contains information about themselves. The information that is typically included in a user's profile varies from community to community, but it often includes the user's name, age, gender, geographic location, and interests. Some online communities also allow users to upload a profile picture.

Profile information is usually visible to other users and can be used by researchers to better understand the user's perspective. In some online communities, such as Facebook, the information in a user's profile is only visible to people who are friends with that user. In other online communities, such as most discussion forums, the information in a user's profile is visible to everyone. When interested in profile data, it is recommended to choose an online community with publicly available user profiles.

However, it should be noted that mining and analysing personal information, such as data from user profiles, has ethical limitations. When conducting research on online communities, it is particularly important to take steps to protect the privacy of the users studied, as, in most cases, it is not feasible to obtain consent from all the users observed online. This article will also discuss ethical considerations in more depth later on. However, one simple way to ensure that their privacy is protected is to anonymise all user data and use IDs instead of usernames. Although doing so would make it impossible to link the data back to a specific individual with all their personal details, it would still allow for community-wide analyses, such as general descriptives, social network analysis, or topic modelling.

**Table:** 

**APPLICATION**When performing social network analysis, it is often useful to focus on a specific group of users. For instance, when studying online health communities, we might focus on users who have been diagnosed with a certain illness and thus contributed to a specific category, allowing us to better understand the behaviour of this specific group of users.

## Data collection

Once the data have been collected, it can be analysed in several different ways. In this section, we will discuss two methods that are commonly used when studying online community data: social network analysis and text analysis.

### Social network analysis (SNA)

Social network analysis helps to understand the relationships between individuals in a social network and what roles individuals may play within a larger group. In online communities, the nodes are usually users, and the edges represent the relationships between them. For instance, in an online discussion forum, the nodes would be the users, and the edges would represent the replies that they have made to each other.

**Table:** 

**APPLICATION**When performing social network analysis, the first step is to construct a social network. First, the relationships must be defined using an edge list or adjacency matrix. In an edge list, each row corresponds to a single edge, with the first column containing the ID of the source node and the second column containing the ID of the target node. There may be more than one edge between two of the same users. Adjacency matrices are similar to edge lists, but instead of representing each edge as a single row, each row and column of the matrix corresponds to a node and each cell represents an edge. The cells in the matrix are either dichotomous (0/1) or continuous. For example, in a discussion forum, a weighted matrix might contain the number of replies that each user has made to every other user.

#### Descriptive statistics of interaction data

Descriptive statistics allow us to explore user interaction patterns within online communities as a whole. For example, evaluating means and distributions of like or reply counts can help researchers see how often individuals interact with each other or what kind of information they like. As such, examining community-wide activity levels can aid in identifying a change in users joining or leaving the community, as well as when and in what categories or threads users are most active. Therefore, these summary statistics can be used to generate and (partially) validate hypotheses about online behaviour.

#### Individual-/dyad-level network measures

Once the social network has been constructed, several different analyses can be performed on it. Generally, social networks can be analysed at three levels: individual connections between users; relationships between groups of nodes in the network; and the overall structure of links in the network.

The most common type of individual-level analysis is centrality analysis, which is used to identify the most important nodes in a social network [11]. There are several different measures of centrality that can be used to identify influential users in different ways, such as degree, betweenness, and closeness centrality.

Degree centrality is a measure of the number of connections a node has to other nodes in the network. However, although a user with high degree centrality may be well connected to others on the network, degree centrality does not indicate the relative importance of those people, as it only considers the number and not the quality of the relationships [7].

Betweenness centrality measures the number of shortest paths that pass through a node. It identifies users who are located in the middle of many different paths and are therefore more likely to be able to control the flow of information between people in the network [12].

Closeness centrality measures the average length of the shortest path between a node and all other nodes in the network. Previous studies have found that people who are close to many other individuals on the network are able to access information more quickly than those who must ’go through’ more people before reaching their person of interest [6,7].

**Table:** 

**CHALLENGE**As there are multiple measures of centrality, an indicator of importance, researchers should consider the prior literature and their theoretical framework to determine which measure or combination of measures to choose. To do so, many comprehensive overviews of the different measures can be consulted, such as those of Brass and Borgatti [7] and Das et al. [11].

#### Group-level network measures

Group-level social network measures are used to analyse the relationships between groups of users in a social network. The most common type of group-level analysis is clustering, which is used to identify natural groups of users in a social network. Therefore, it can be used to identify patterns in the way that people form groups with others in the network.

There are several different algorithms that can be used to perform clustering, such as k-means and hierarchical clustering. K-means is a simple algorithm that groups nodes according to their proximity to other nodes in the network. Hierarchical clustering is a more complex algorithm that groups nodes together based on their shared connections with other nodes in the network.

Both k-means and hierarchical clustering can be used to identify groups of users that are similar to each other in terms of their online behaviour. For example, if we were interested in identifying groups of people who are likely to have similar knowledge and experiences, we could use clustering to group together users who post in the same online forums or who follow the same people on Twitter.

Another type of group-level social network analysis is community detection, which is used to identify groups of nodes that are more densely connected to one another than they are to other nodes in the network. Community detection can be used to identify online communities, such as the members of a forum who are most active in discussions and, therefore, form the ’social core’ of the whole community.

**Table:** 

**APPLICATION**There are a number of different algorithms that can be used to perform community detection, such as the Girvan-Newman algorithm and the Markov Cluster algorithm. The Girvan-Newman algorithm is a widely used community detection algorithm that works by removing edges from the social network until the network is broken up into several smaller communities. The Markov Cluster algorithm is a newer community detection algorithm that works by identifying the communities in a social network that are most tightly interconnected.

#### Network-level network measures

The most common measure of overall network connectivity is density, which measures the number of connections that exist in a social network relative to the maximum number of possible connections. Density can be used to compare different online social networks or to compare the same social network at different points in time. For example, if we wanted to know whether people are more or less connected to one another in one sub-community than in another, we could compare the density of those communities.

Average path length is a measure of the distance between two nodes in a social network. It is calculated by taking the sum of the distances between each pair of nodes in the network and dividing it by the number of pairs of nodes. Average path length measures the distance, that is, the number of steps, between any two nodes in a social network, taking the sum of the distances between each pair of nodes and dividing it by the number of pairs of nodes. This measure is another indicator of the efficiency of the information flow in a social network. The lower the average path length, the more efficient the flow of information in the network.

#### Network visualisations

A social network can also be visually represented as a graph, with the nodes represented as circles and the relationships as lines between the nodes. Social network visualisations provide a map of all the connections between nodes in a network which can be used to understand patterns in behaviour within that community, such as who replies to whom and what topics they are contributing to. Network visualisations can also help spot outliers, unusual data points, and patterns that might not be evident from looking at the raw data.

Although a well-structured graph may be much more impactful in communicating findings than any number of words, especially large networks, can be difficult to study visually. Thus, while social network visualisations can aid interpretation and provide insight, it is in combination with (statistical) analysis that visualisations give us strong grounds for inference and/or conclusions. On their own, visualisations should be seen as an exploratory and presentational device.

**Fig. 3:**
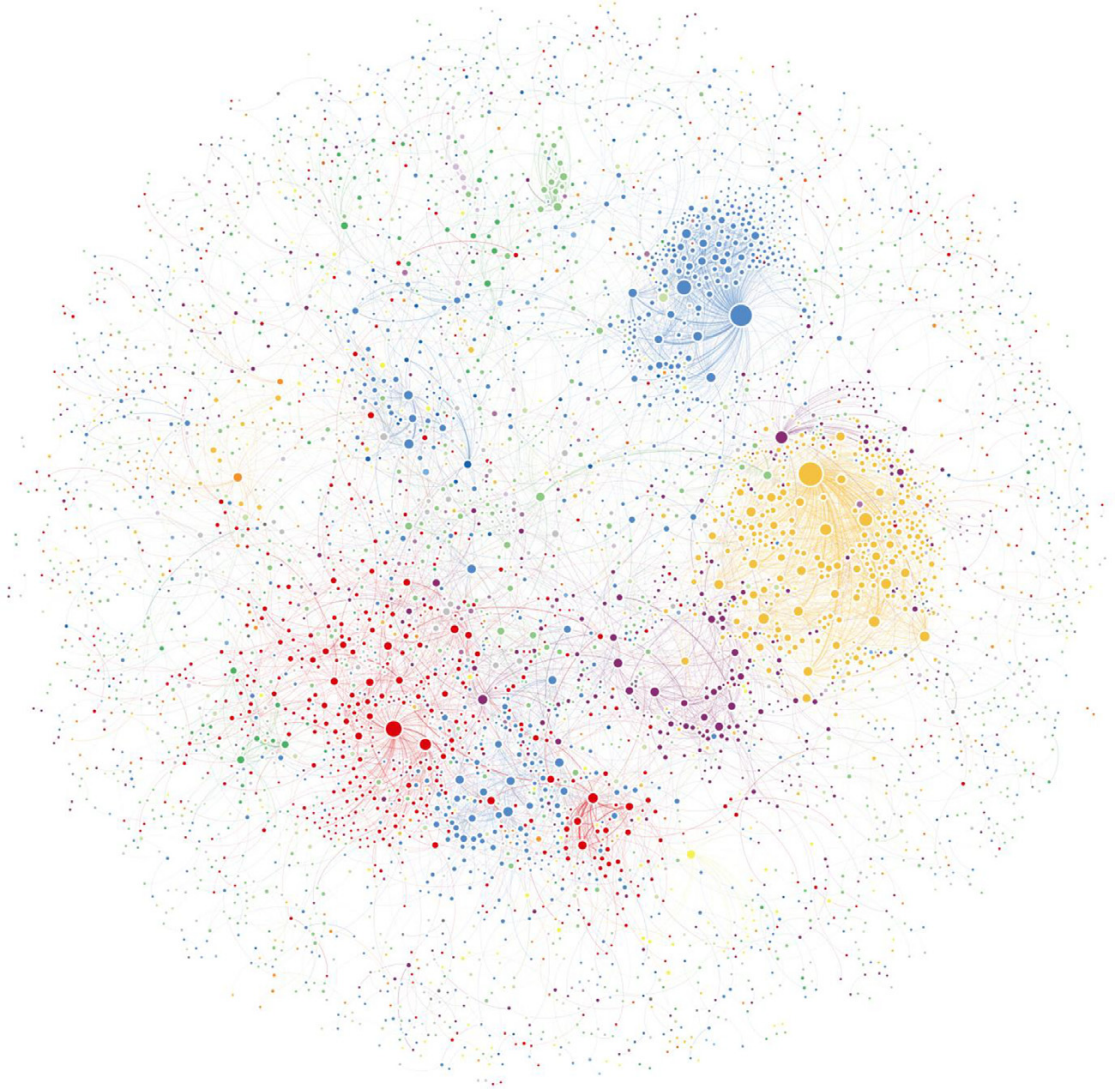
A social network visualisation of an entire online health Q&A community at one point in time [28]. The colours represent each thematic category (e.g., yellow for ’Mental Health’), while the size of the nodes indicates the degree centrality of each individual displayed.

Fig. 3 shows an example of a social network visualisation of a whole online health community. The community is a discussion forum with 32 main categories where users can post questions and answers about health concerns. The nodes in the network are the members of the forum, and the lines connecting them represent replies between members.

The size of the nodes is proportional to the number of posts each user has made on the forum. The colours of the nodes represent the main category to which each user has contributed predominantly. For example, a user who has posted a total of five replies, with three of them in the ‘Mental Health’ category and two in the ‘Brain’ category, would be coloured yellow. The thickness of the lines represents the frequency with which the members interact with each other. For example, the thicker lines connecting members in the ‘Mental Health’ category (yellow) indicate that these members interact with each other more often than members in other categories.

### Content analysis: words used

In online social networks, people often not only describe information, but also express their opinions on various topics. This creates a rich source of data that can be used to understand people's views and opinions on different issues. By analysing text posted in online communities, researchers may be able to evaluate three different aspects: objectivity, subjectivity, and personality.

#### Objectivity

First, the words used may indicate the ’objective’ value of the post. For example, a post that uses more complex or domain-specific words such as health-related terms in an online health community is more likely to bring informative value to its readers than a short and simple post that does not use such domain-specific words.

**Table:** 

**APPLICATION**To be able to judge the ’objective’ value of a certain post, it is important to find or, if necessary, create a dictionary of terms that are considered complex or domain-specific. For many subject areas, such as medicine or information technology, crowd-sourced dictionaries or word lists developed by researchers are available.

#### Subjectivity

Second, the words used can provide information about the affective state of the author of the post. They may give some indication of the user's subjective experience and the meaning he or she is conveying. For example, a post that uses phrases like ‘I feel’ or ‘I think’ is likely to be more subjective than a post that describes less subjective feelings, thoughts, and observations.

**Table:** 

**APPLICATION**Software tools such as *LIWC* are commonly used to assess the use of certain pronouns and words indicative of certain emotions and cognitive processes (Pennebaker et al., 2001; [32]). Furthermore, R offers access to a variety of sentiment dictionaries such as *nrc* and *bing* with which words with an emotional sentiment can easily be identified.

#### Personality

Finally, the use of certain words can be a marker of the user's identity. Previous studies have linked the use of certain words and pronouns with different types of personality [29]. For example, the use of singular first-person pronouns (e.g., ‘I’, ‘me’, and ‘my’) has been found to be positively correlated with neuroticism and extraversion, while the use of plural first-person pro-nouns (e.g., ‘we’, ‘us’, and ‘our’) has been found to be positively correlated with agreeableness and conscientiousness [20]. In addition, some personality traits are also likely associated with certain emotions. For example, previous studies such as that of Barlow et al. [3] have found the personality trait neuroticism to be negatively correlated with the tendency to experience positive emotions (i.e., people who are high in neuroticism experience fewer positive emotions) and positively correlated with the tendency to experience positive emotions (i.e., people who are high in neuroticism experience more negative emotions).

**Table:** 

**POTENTIAL FUTURE RESEARCH**When analysing the formation and evolution of social relationships in different settings, a wide range of questions could be asked about possible factors that might play a role in influencing who connects with whom. These questions could relate to three different levels: (1) entire network with a certain ’atmosphere’, (2) relationship between two people with certain characteristics, or (3) individual aspects such as personality. One may choose to explore, for instance, to what extent individuals with similar or different personality characteristics connect with one another.

### The role of time

One of the main advantages of analysing online community data is the ability to conduct longitudinal analyses. Longitudinal studies allow researchers to explore how the network changes over time. This is particularly useful for understanding social phenomena that unfold over extended periods of time, such as the development of relationships, or how new behaviours and social norms emerge and spread throughout a community.

Furthermore, longitudinal data can be used to simulate the co-evolution of individual and group behaviour and network structure. In other words, by taking into account the observed changes in both the network structure and the behaviour of individuals, such as their attitude towards a particular topic, we can generate hypotheses about how a certain behaviour affects the forming and discontinuing of certain network ties and vice versa. RSiena is an R package commonly used to create these types of stochastic actor-oriented models that simulate how social networks and behaviour influence each other [30,31].

**Table:** 

**POTENTIAL FUTURE RESEARCH**Especially the co-evolution of network and behaviour dynamics is a promising avenue for future research. Such longitudinal analyses allow us to infer causality and better understand contextual and personal factors that can shape the formation of social networks, as well as individual and group behaviour.

## Ethical considerations

When conducting research on social networks, and on online networks in particular, it is important to consider the ethical implications of collecting and analysing data [1,34]. In online communities, people often share sensitive and personal information. Especially, some user groups, such as online health communities or communities involving children and young adolescents, are vulnerable. Misinformation or online bullying in these communities can have significant negative consequences for the health and well-being of participants, as well as the validity of the study.

Researchers must be careful not to violate the privacy of participants in their study. One way to do this is to ensure that all data collected are anonymised and that no individual can be identified from the data. Additionally, researchers should seek the approval of an institutional review board before conducting their study.

**Table:** 

**CHALLENGE**The ethical considerations of online data collection have gained increased attention in recent years from researchers and the general public. While there are some guidelines on how to ethically collect online data, these vary per institution and area of research, and as such often do not sufficiently address key issues such as transparency and consent. To address these concerns, there is a need for more comprehensive and consistent guidelines on ethical online data collection.

### Obtaining consent

Normally when collecting data from individuals, it is important to obtain the consent of the study participants. However, in the case of most online communities, consent cannot be obtained because it is not feasible for the researcher to contact every individual who participates in the community. In some cases of very small and targeted studies, researchers may be able to contact the administrators of the online community and ask them to distribute a consent form to all members of the community. In other cases, researchers may be able to contact participants who have shared particularly sensitive information and ask them for their consent to use this information in the study.

Even if the data is anonymised to avoid re-identification, it is advisable to seek approval from the operators of the online community at hand before collecting and analysing the data. Although public data that is visible online without the need to log in and agree with any terms of use is generally considered to be in the public domain, it is still desirable that researchers respect the rules of the community. This means being mindful of any terms of use that exist for the platform, such as certain user information that should not be extracted, as well as any specific guidelines for research that may be provided by the administrators [33]. Depending on the specific protocols, seeking prior approval from the community might also be a necessary first step when seeking approval from Institutional Review Boards for a particular research project involving online network data.

### ‘Spying’ on public information

Many online communities can be accessed without the need to register a user account. Then, as noted above, collecting data without registering a user account does not constitute a violation of the terms and conditions of the community. However, researchers should be aware that there is no guarantee that all content in an online community will be accessible without registering a user account, so some information may remain inaccessible. Furthermore, users may not be aware that the information they post becomes part of the public domain and that they may be ”spied” on for research purposes. For instance, in an exploratory survey of Twitter users, Fiesler and Proferes [14] found that most users were unaware that their public tweets could be used by researchers and do not agree with their tweets being used without their consent. The authors argue that while these assumptions and attitudes vary depending on the context and type of study, the users’ autonomy and fear of re-identification should be respected as much as possible.

### Researcher participation

Lastly, potential participation of any researcher in online communities may have profound ethical implications. To avoid intrusion, both from a research method and research ethics point of view, researchers must be transparent about their identity and affiliation and should not disguise themselves as members of the community to collect data. Additionally, they should always avoid taking advantage of the trust that members of online communities are often willing to give to others, or avoid any accusations of such conduct.

## Limitations and future improvements of the proposed method

The method proposed in this article has several limitations that should be considered when conducting online social network research.

### Software compatibility

First, some researchers may prefer to use software packages and file formats other than those described in this article. The method we propose is based on the use of R and Gephi, which some researchers may not be familiar with. However, these software packages are relatively user-friendly or, if needed, could be substituted with other software packages without too much difficulty as many of the steps proposed are similar, regardless of the software package used. In particular, the strategic considerations for choosing a suitable data source and analysing text and relationships found in online communities are universally applicable.

### Collecting real-time data

Second, our method is focused on the one-time collection of data from online social networks. While online communities are constantly changing, the method proposed in this article can be used to collect and analyse a snapshot of the online social network at a particular point in time. This limitation can be overcome by conducting multiple data collections over time and then analysing the changes in the online social network over time. In this way, researchers can answer questions about how social networks change over time. For example, one may choose to collect online social network data on a weekly basis over multiple months or years to answer questions about how social structures and influences develop over time. The most important adaptation was to write the code so that it could be run weekly without requiring too much manual intervention. For example, by automating the merging of previous and new observations, the filtering of duplicates, and the text processing, the time required to perform the online social network analysis was reduced from several hours to only a few minutes.

**Table:** 

**RECOMMENDATION**The proposed method can be used to collect and analyse a snapshot of the online social network at a particular point in time. However, by automating parts of the workflow, such as the merging and filtering of data, and the text processing, which likely remains similar over time for the same data source, the time required to collect data continuously can be reduced dramatically.

### Text-based online communities

Third, our method is mostly suited for the analysis of text-based online communities such as online discussion forums. While text-based social networks account for a large majority of online networks, there are some that are image-based or video-based. Furthermore, some technical design choices made by online community operators, such as continuous loading of new information as a user scrolls through content, make scraping the data more difficult. As explained in subsection ’Selecting an Online Community’, researchers should consider the type of online social network data they require when selecting an online community for data collection and adapt the method to fit the chosen data source.

### Alternative methods for analysing social networks

Fourth, our method does not fully take into account the potential use of different types of social network modelling. For example, the interaction data collected with this method could be used as input for stochastic actor-based modelling (RSiena) to analyse network and behaviour dynamics or for ego networks. Instead of analysing the social network as a whole, ego networks consider only a portion of a social network formed around a focal node (’ego’) and its direct ties with other nodes (’alters’). This may be particularly helpful in scenarios where researchers are interested in studying specific users, such as highly active outliers or otherwise influential users.

### Addressing outliers

Finally, these outliers present a challenge for the analysis of social networks online. In particular, the decision to remove or keep them can have a significant impact on the results of social network analysis. One method to deal with outliers is to use the trimmed mean, which is the average of a set of data after removing the largest and smallest values. This method has been shown to be effective in the context of online social networks [36]. However, it is important to note that the use of a trimmed mean can introduce bias into the results of social network analysis, as it can make the data appear more clustered than it actually is.

Furthermore, by removing outliers to make the data more normally distributed, potentially interesting insights about the behaviour of the most active and influential users are lost. Particularly in online discussion forums, a very small number of users known as ’power users’ often account for the majority of the content and might therefore affect the validity of the results. For example, a study of anti-vaccine misinformation during the covid-19 pandemic showed that 65% of all anti-vaccine misinformation on social media was shared by just 12 accounts, the so-called ’Disinformation Dozen’ [8]. Therefore, researchers should carefully weigh the implications of removing outliers with the potential need for nonparametric statistical analyses.

**Table:** 

**RECOMMENDATION**Power users are commonly found as outliers in online communities. Although they may affect the validity of the results, they may also offer insight into the dynamics of not only those users, but the community as a whole. Therefore, the decision to remove outliers as is commonly done for statistical analyses should be made carefully.

Despite these limitations, the method proposed in this paper provides a valuable starting point for online social network research. Additional methods that take into account other possible structures of online communities, real-time data collection, and more effective ways of analysing outliers should be developed in the future to allow for a more comprehensive analysis.

## Conclusion

Online communities provide a valuable source of data for researchers interested in studying individual and group behaviour. While online social network research is growing, many questions have yet to be explored. This article provides a method for researchers who are interested in studying online social networks but do not have a background in Computer Science or other technical disciplines. The method is based on the collection of interaction data using a web scraping and social network analysis approach. Although this method has several limitations, it is a valuable starting point for online social network research.

When mining and analysing online social network data, there are a few important considerations that researchers should keep in mind. First, online communities can be a valuable source of data for understanding human behaviour if the available data are well aligned with the objectives of the study. By analysing the content of posts in a social network, researchers can learn about the emotions that people are experiencing, their opinions and behaviour, and how they interact with others.

Second, online social networks are a valuable resource for conducting longitudinal studies. Longitudinal studies allow researchers to track how the network changes over time and how new behaviours and social norms emerge and spread throughout a community. Even more so, online social networks can be used to simulate the co-evolution of individual and group behaviour and network structure. By taking into account the observed changes in both the network structure and the behaviour of individuals, researchers can generate hypotheses about how a certain behaviour affects the forming and discontinuing of certain network ties.

Lastly, this method article described a range of considerations and ethical implications that researchers may face when conducting research using online social network data. As with any data source, researchers should take into account the potential for bias in the data and take steps to mitigate this bias. In online social networks, there is a risk of including personal information in the data set without the consent of the individuals involved. Therefore, researchers should ensure that any data they include in their analysis are anonymised and do not contain any identifying information about the participants.

Bearing in mind the considerations and implications discussed in this article, the data of the online communities can be a valuable source of information for researchers who are interested in studying human behaviour. By carefully collecting and studying online social network data, researchers can use our method to answer new and complex research questions about the circumstances and ways in which people interact with each other online.

## Ethics statements

The data of the participants, affiliated to the social media platform, has been fully anonymised and the social media platform's data redistribution policy was complied with.

## Related research article

Rueger, J., Dolfsma, W., & Aalbers, R. (2021). Perception of peer advice in online health communities: Access to lay expertise. *Social Science & Medicine*, 277, 113,117. doi:10.1016/j.socscimed.2020.113117

## CRediT authorship contribution statement

**Jasmina Rueger:** Conceptualization, Methodology, Software, Formal analysis, Investigation, Data curation, Writing – original draft. **Wilfred Dolfsma:** Validation, Writing – review & editing, Supervision, Resources. **Rick Aalbers:** Validation, Writing – review & editing, Supervision.

## Declaration of Competing Interest

The authors declare that they have no known competing financial interests or personal relationships that could have appeared to influence the work reported in this paper.

## Data Availability

Data will be made available on request.
